# The Effect of Vibration on the Acceleration of Wound Healing of Diabetic Neuropathic Foot Ulcer: A Prospective Experimental Study on Human Patients

**DOI:** 10.3390/healthcare11020191

**Published:** 2023-01-09

**Authors:** Sitti Syabariyah, Elly Nurachmah, Budiman Darmo Widjojo, Sabarinah Prasetyo, Hiromi Sanada, Irianto, Gojiro Nakagami, Suriadi, Tutur Kardiatun, Urfa Khairatun Hisan

**Affiliations:** 1 Department of Medical Surgical Nursing, University of Aisyiyah Bandung, West Java 40264, Indonesia; 2 Department of Medical Surgical Nursing, University of Indonesia, Depok, West Java 16424, Indonesia; 3 Faculty of Medicine, Universitas of Indonesia, Jakarta 10430, Indonesia; 4 Faculty of Community Health, Universitas of Indonesia, Depok, West Java 16424, Indonesia; 5 Department of Wound Care Management/Gerontological Nursing, Graduate School of Medicine, The University of Tokyo, Tokyo 113-8654, Japan; 6 Department of General Education, Faculty of Resilience, Rabdan Academy, Abu Dhabi 22401, United Arab Emirates; 7 Department of Nursing, Institut Teknologi dan Kesehatan Muhammadiyah Kalimantan Barat, Kabupaten Kubu Raya 78117, Indonesia; 8 Faculty of Medicine, Universitas Ahmad Dahlan, Yogyakarta 55166, Indonesia

**Keywords:** vibration therapy, diabetic foot ulcer (DFU), diabetic wound, wound care, nitric oxide (NO)

## Abstract

Diabetic foot ulcers are a common complication that occurs in approximately 15 percent of patients with diabetes mellitus. Over 60% of diabetic foot ulcers are caused by underlying neuropathy. Former studies on diabetic animals with foot wounds found that vibration platforms significantly accelerate wound healing by catalyzing epithelization, promoting angiogenesis, and enhancing muscle bulk. This result suggests that there is evidence that vibrations may accelerate diabetic neuropathic ulcer healing in human patients. However, to the best of our knowledge, the effect of vibration on the enhancements of diabetic foot ulcer healing in human patients is rarely investigated. Hence, in this work, we conducted an experimental study with human subjects to investigate whether vibration therapy, as a complement to the standard wound treatment, can accelerate the wound healing rate of diabetic neuropathic foot ulcers. In this prospective experimental study, 80 participants diagnosed with Wagner grades I–III diabetic neuropathic foot ulcers were randomly distributed to experimental (*n* = 40) and control groups (*n* = 40). Patients in the intervention group received standard wound treatment and vibration wound therapy (VWT), whereas patients in the control group retrieved only standard wound treatment. The results (*p* = 0.024, *α* = 0.05) show notable differences in the median healing rate between the intervention group (25 days, 95% CI: 20.3–29.7) and control group (33 days, 95% CI: 25.6–40.4), with the effect-size *r*, Cohen’s *d*, Glass’s Δ, and Hedges’ *g*, respectively, being 0.810, 2.764, 2.311, and 2.772. Moreover, the nitric oxide (NO) level, wound closure area, and wound healing score after intervention significantly differed between the two groups (*p* < 0.05), putting the intervention group on a higher level than the control group. Furthermore, positive associations were found between the NO level and wound healing closure rates. These findings suggested that VWT enhances diabetic neuropathic foot ulcer healing in terms of healing rate, wound closure area, healing score, and elevated NO level. Considering that no clinically adverse effects were found in the patients induced with vibration intervention, VWT can be regarded as a complementary therapy to the existing ones to accelerate the healing of DFUs.

## 1. Introduction

Diabetic foot ulcers are among the most common complication of diabetes mellitus [1]. It is approximated that diabetic foot ulcers occur in about 15 percent of patients with diabetes [2]. Among the 451 million people diagnosed with diabetes in 2017 [3], it is estimated that there are more than 67 million diabetes mellitus patients with diabetic foot ulcers. Moreover, the number of people with diabetes is expected to rise to 693 million in 2045 [3]. Indeed, 67 million diabetic foot ulcer patients itself is already a serious number, especially when considering that diabetic foot ulcers are among the most frequent causes of patient disability and morbidity [4]. It is found that more than 85% of lower-limb amputations in diabetic patients are preceded by foot ulceration [5]. This evidence suggests that a proper and decent prevention-and-treatment of foot lesions is critical [6,7,8]. Study [9], which studied 644 subjects with a cumulative incidence of 26.6% for diabetic foot ulcers, 5.8% for lower extremity amputation (LEA), and 14.0% for death, revealed that diabetic foot ulcers are more than just a marker for complication status. Diabetic foot ulcers are significantly responsible for the LEA and death incidence of diabetic patients.

This study was conducted in Indonesia due to its high prevalence of diabetes patients. In fact, Indonesia ranked seventh in the number of diabetes patients worldwide, with 10.7 million patients in 2019 [10]. In 2030 and 2045, this number is estimated to rise to 13.7 and 16.6 million, respectively [10]. To give an illustration, in 2007, at Cipto Mangunkusumo National General Hospital located in Jakarta, Indonesia, about 33% of diabetes patients were hospitalized due to DFU, where these patients were associated with higher treatment costs and a longer average length of stay [11]. A study published in 2005 shows that the average hospital stay of patients suffering from DFU is 45.3 days, costing an average of more than IDR 1.6 million for each patient. In 2005, the minimum regional salary for Jakarta (where the hospital is located) was IDR 819,000, which is about half of the required cost of a DFU patient [12]. Furthermore, among these DFU patients, the amputation rate was 32.5%, inevitably reducing the patient’s quality of life [13,14].

Diabetic foot ulcers typically result from underlying neuropathy, peripheral vascular disease, microcirculatory damage, or bad glycemic control [15]. Over 60% of diabetic foot ulcers are caused by underlying neuropathy [16,17]. The manifestations of neuropathy in affected individuals are the consequence of metabolic problems brought on by hyperglycemia. The production of myoinositol, which is necessary for regular neuron transmission, is decreased due to the buildup of sugar products in nerve cells. The chemical conversion of glucose causes the nerve cell to undergo more oxidative stress and vasoconstriction, which will cause ischemia and encourage nerve cell destruction and death. The formation of ulcers is caused by peripheral neuropathy’s sensory loss. Patients frequently lack the ability to recognize the injury to their lower extremities because trauma happens in the affected region [18].

Impaired wound healing is a prominent characteristic of diabetic foot ulcers [19]. Rational and effective treatments can prevent ulcer deterioration, accelerate healing, and reduce the amputation rate [20]. The standard diabetic foot ulcer treatment principles comprised ulcer protection and relief of pressure, infection prevention and treatment, local wound care, skin perfusion restoration, recurrence prevention, comorbidities treatment, and metabolic and diet control, followed by patient and family education/motivation [21,22,23,24]. There are already numerous medical treatment options available for diabetic foot ulcer care. However, the wound closure and recovery rate remain slow [25]. Hence, many researchers are vying to develop new methods to enhance diabetic foot ulcer healing and recovery [23], including vibration-assisted diabetic wound healing [26,27,28].

A previous study on 96 diabetic rat models with foot wounds shows improved circulation in the group exposed to a low-magnitude high-frequency vibration intervention [26]. The improvement is evaluated by micro-CT angiography, histology, and doppler USG. It was also found that there was an improved bone formation and mineralization in the vibration group. In addition, vascular endothelial growth factor (VEGF) that catalyzes hematopoiesis, bone formation, wound healing, and angiogenesis was significantly increased in the mentioned group. Subsequently, the study revealed that vibration platforms significantly increase the wound healing rate and enhance skin microcirculation with the upregulation of healing markers. In the group induced with vibration intervention, it was found that the wound size was significantly smaller and that the perfusion in the wound was substantially better compared to the control group.

For human patients, vibration intervention has been employed in medical rehabilitation, neurology, physiotherapy, and orthopedic surgery. In recent years, however, there has been an increase in vibration therapy research for other clinical applications, especially in wound and ulcer healing [29,30,31,32,33,34]. In [32], it is shown that vibration helps the healing of Stage I pressure ulcers in elderly patients. However, in open and deeper ulcers, [35] revealed that low mechanical vibration might improve the healing of grade A1 diabetic polyneuropathy foot ulcer. Grade A1 is described as a superficial wound into the dermis. Further, in [33], the authors found that a surface acoustic wave (SAW) vibration may enhance cell growth and accelerate the wound healing speed by up to 135 ± 85%. In [34], the author revealed that using direct skin vibration improves the skin blood flow in a notable manner.

However, despite previous results suggesting that vibrations may accelerate diabetic ulcer healing in human patients, the number of practical validations investigating the effect of vibration on enhancements of diabetic neuropathic foot ulcer recovery in human patients remains far from sufficient. Thus far, no strong evidence supports that vibration can enhance diabetic neuropathy wound healing in human patients. A study in [35] investigated the effect of mechanical vibration therapy on the healing of foot ulcers in 29 diabetic polyneuropathy human patients. The result suggests that low mechanical vibration may enhance diabetic foot ulcer healing.

In our preliminary study [36] with 31 patients suffering from diabetic neuropathic foot ulcers, we found that vibration therapy, as a complementary treatment, significantly increases the healing speed of diabetic neuropathic foot ulcers. However, both works lack a sufficient number of patients, making it insufficient to generalize the result, let alone justify the wide use of vibration therapy for diabetic neuropathic foot ulcers in the clinical setting. Moreover, our interim study [36] lacks deep analysis. Therefore, in this work, we conducted a follow-up study with a larger number of participants and sufficiently better clinical analysis as an effort to contribute to the currently insufficient validation of the vibration therapy effect toward diabetic neuropathic foot ulcer healing. Fortunately, previous efforts using vibration interventions proved that such interventions are safe and provide no negative effects for human subjects [32,33,34,35,36,37,38,39]. Lastly, the outcome of our study was evaluated using the following parameters: healing rate, healing score, nitric oxide (NO) level, and reduction in wound size over the 12-week intervention period.

In summary, the main contributions of this paper are two-fold:

1. We propose complementary diabetic neuropathic foot ulcer wound care for diabetic patients using vibration therapy to enhance the wound recovery rate and accelerate the wound closure speed.
2. In this work, we evaluated the effect of vibration intervention on NO level prior to and after the intervention. The literature shows that NO is a substantial factor in catalyzing wound healing.

Finally, the remainder of this article is organized as follows. We present the materials and methods, including the vibration therapy setup and intervention, medical assessment, and statistical analysis in Section 2. In Section 3, we present the result of this study, followed by a discussion in Section 4. Section 5 presents the limitations of this study and recommendations for future works.

## 2. Subjects and Methods

This prospective study was conducted in two general hospitals in Indonesia: Ade Muhammad Djoen General Hospital, Sintang, and Sudarso General Hospital, Pontianak. Both hospitals are located in the West Borneo Province of Indonesia. The protocol of this study was approved by the Ethical Committees of Clinical Research (ECCR) from both hospitals. Prior to study enrollment, all participants provided their written informed consent. We enrolled patients that met the following inclusion criteria: age ≥ 18 years and neuropathic foot lesions with Wagner grades I–III. We excluded patients with ischemic ulcers, peripheral vascular disease, osteomyelitis, widespread gangrene with unavoidable lower-limb amputation, high-severity comorbidities, hypoalbuminemia (<2.5 g/dL), renal failure requiring hemodialysis or peritoneal dialysis, or uncontrolled hypertension (systolic > 160 mmHg, diastolic > 100 mmHg). These criteria are designed in such a way that situational bias can be avoided.

In this study, we report 80 cases of Wagner grades I–III diabetic neuropathic foot ulcers. Eligible participants were randomized into experimental (n=40) and control (n=40) groups. The intervention group consisted of participants who received standard care and complimentary vibration therapy, whereas the control group received only standard care. We then evaluated wound size, healing score, and NO level in these patients over a 12-week experimental period.

### 2.1. Vibration Therapy Setup

**Figure 1:**
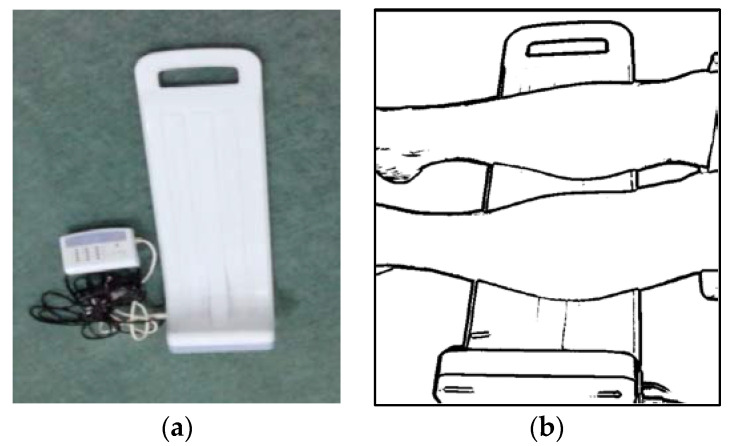
(**a**). RelaWave^®®^ Matsuda Micronics Vibrators: The blue (bottom) part produces vibration, and the white (top) part distributes the vibration. (**b**). Vibration therapy setup.

The vibration interventions were conducted using RelaWave^®®^ Matsuda Micronics Vibrators (see Figure 1a), supported by the University of Tokyo, Tokyo, Japan. This vibrator weighs 2000 g, has dimensions of 616 × 182 × 144 mm^3^, and is equipped with a controller to set its amplitude and frequency. The amplitude modulation cycle and the vibrator frequency, respectively, were set to 1.78 m/s^2^ and 47 Hz following our preliminary study [36]. These values are within the recommended occupational exposure limits of vertical and horizontal vibration acceleration at a vibration time of 15 min and a frequency of 50 Hz, which are ≤ 13.2 m/s^2^ and ≤ 37.5 m/s^2^, respectively [32].

### 2.2. Vibration Therapy Intervention

The vibration therapy was conducted in a thermoneutral room with the environmental temperature ranging between 24 and 29 °C. To prevent situational bias, regardless of their study group (i.e., intervention group or control group), all participants retrieved similar standard diabetic neuropathic foot ulcer wound care recommended by the Indonesian Ministry of Health. The standard diabetic neuropathic foot ulcer wound care comprises adequate off-loading, debridement, and careful daily ulcer monitoring. However, patients in the intervention group received vibration adjuvant therapy with a frequency of 47 Hz for 15 min after standard wound care every two days during the 12-week study period. As illustrated in Figure 1b, the lower leg of the participants is placed on the top of the vibrator. A rubber pad was inserted between the leg and the vibrator to prevent skin irritations. According to the wound characteristics, the dressing was changed if required (approximately 3–4 times every week) using standardized saline-moistened gauze.

### 2.3. Medical Assessment

When a first-time patient arrived, patient demographics, medical records, vital signs, wound and amputation history, diabetes and wound duration, BMI, wound location, ulcer size, and ulcer grade were recorded. Additionally, any kind of drugs previously (or routinely) taken by patients, either related to diabetes or other diseases, were noted to prevent possible outcome interference. Monofilament test, HbA1c, Albumin, and NO levels were also conducted before, during, and after the intervention. During the study period, every medical intervention and drug intake (other than the designated ones) was carefully monitored to prevent outcome bias. Lastly, the patients’ wounds were periodically monitored and evaluated using observation sheets and photography devices. The wound area was measured using a wound ruler and a transparent film scale. The measurement was conducted by the same nurse for all participants during the study period to prevent interperson subjectivity. The area was calculated based on the ellipse shape formula using the major and minor diameters of the wound. This calculation was then validated using vision-aided wound area measurement. The photograph of the wounds was taken after the debridement process and before the dressing application. The relative wound reduction (%/day) was calculated by $\left(\left(\mathrm{initial}\;\mathrm{areas}-\mathrm{final}\;\mathrm{areas}\right)\;\mathrm{initial}\;\mathrm{areas}\;\mathrm{examination}\;\mathrm{period}\times 100\right)$. The delta wound area reduction (mm/day) was obtained by dividing the measurement difference by the number of observation days. These reports then became the primary outcomes for evaluating the effectiveness of the proposed vibration therapy.

### 2.4. Statistical Analysis

The Wilcoxon signed-rank test was used to compare the outcomes between the two groups of study statistically. The Mann–Whitney test was used to compare the average difference between groups. Kaplan–Meier curves were used to assess the wound healing rate between the intervention and control groups from the beginning of the study to the end of the study. In this study, we defined a completely healed wound as a closure of the wound surface with no discernable exudate and without drainage. This definition is in line with the ones in the previous study [40]. The Cox proportional hazards regression model was used to calculate the relative risks for healing incidents in the vibration therapy group against the control group by factoring in the vibration treatment. All *p*-values reported were two-sided, with *p*-values <0.05 considered statistically significant.

## 3. Results

**Figure 2:**
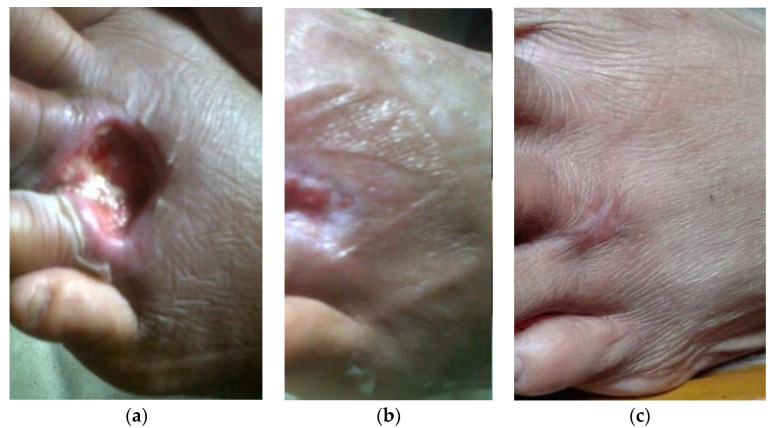
An ulcer photograph of a patient (age = 49) located on the left foot’s central aspect. This ulcer had remained open for one week. The patients received a standard diabetic neuropathic foot ulcers treatment and a complementary VWT intervention. (**a**). Day 0 before treatment (wound area = 397.2 mm²). (**b**). Day 27 (wound healing stage). (**c**). Day 28 (the wound is completely healed).

Table 1 presents the characteristics of patients included in this study. In this study, we excluded patients that are clinically diagnosed with ischemic foot ulcers. As in Table 1, all participants have an ABI level of ≥ 0.8, supporting the clinical diagnosis that all the patients had a 0 grade regarding ischemia level [41]. As depicted in Table 1, there were no significant demographic differences between the vibration wound therapy (VWT) and control treatment groups (e.g., age, sex, BMI, and wound and amputation histories). There were also no notable differences in diabetes and wound durations, wound location, and baseline ulcer size. However, it was found that the patients induced with VWT had a shorter median healing rate of 25 days (95% confidence interval (CI), 20.3–29.7 days), while the subjects of the control group had a longer median healing rate of 33 days (95% CI, 25.6–40.4 days). Unfortunately, there was one patient in the control group whose wound was not healed within the designated 12-week study period. Therefore, this patient was excluded from further analysis. Nevertheless, a statistically significant difference (α=0.05) between the median of both groups was obtained by the Wald test, with the *p*-value of *p* = 0.024 (see Table 2). In Table 3, we present the calculated size effect for each group. In Figure 2, we present the sequence of the ulcer healing stage of a patient receiving complementary VWT intervention. In this study, we also calculated the hazard ratio (HR) and found that the wound healed 1.69 times faster in the vibration intervention group than in the control group (95% CI of the HR, 1.07–2.68). Table 4 displayed the wound closure area between the two groups. It was found that there were notable improvements in the relative area reduction (%/day) and delta reduction (mm/day) of the wound closure in the vibration group compared to the control one. Lastly, there were no reported negative effects on the patients with VWT intervention, suggesting that the performed intervention was adequately safe for the human patients.

The Wilcoxon test was used to compare the NO-level differences before and after intervention between groups. This test is based on the Shapiro–Wilk normality test, and the obtained data distribution was abnormal. Every NO-level measurement was conducted twice, and, as shown in Table 5, there were no differences in the NO levels between the intervention group and control group before intervention (p=0.502). However, the two groups observed significant differences in the NO levels postintervention. The average NO levels after intervention were significantly higher in the vibration intervention group (2.91 mol/mL) compared to the control group (0.86 mol/mL), with a *p*-value of <0.001.

The Mann–Whitney test was used to compare the average difference between groups in the delta NO level (the difference between the NO levels before and after intervention). As in Table 5, it was found that there is a notable difference in the delta NO value between the control group and the VWT group (p<0.001). The control group sees a slight degradation in the NO levels, whereas the VWT group experiences a significant increase in NO levels. The degradation in NO levels in the control group is in accordance with the finding of the previous study, revealing that aging and diabetes can decrease the NO synthase or decrease the bioavailability of NO [42]. As for the increasing NO levels in the VWT group, this result indicates that the VWT may catalyze the NO formation. These phenomena are further discussed later in this manuscript.

**Figure 3:**
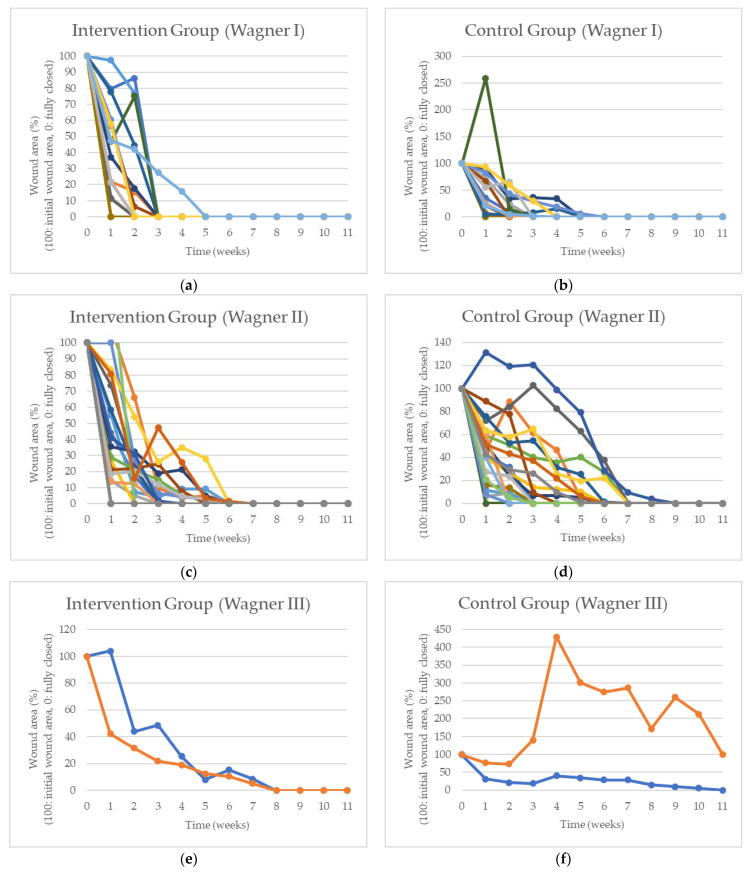
Graphs of wound area for the intervention and control groups. The wound area is measured once every week for 12 weeks. Each line corresponds to the healing rate of each patient. (**a**). Intervention Group (Wagner I). (**b**). Control Group (Wagner I). (**c**). Intervention Group (Wagner II). (**d**). Control Group (Wagner III). (**e**). Intervention Group (Wagner III). (**f**). Control Group (Wagner III).

Figure 3 depicts the measured wound area for the intervention and control groups for patients in each Wagner grade. The wound area is measured once every week for 12 weeks. Each line in Figure 3 corresponds to the healing rate of each patient. As in Table 1, each group (i.e., intervention and control) comprises 17 patients with Wagner I grade, 21 with Wagner II grade, and 2 with Wagner III grade.

Unfortunately, the wound of one of only two patients with Wagner III grade ulcers in the control group failed to fully close within the 12-week study period. Although we examined the patients’ initial conditions and ensured that there was no severe infection that might alter the outcomes and/or produce biased results at the beginning of the study, as time went by, one of the patients (Wagner III) in the control group suffered from advanced infection, contributing to the factors that caused their wound to fail to fully close within the 12-week designated period.

**Figure 4:**
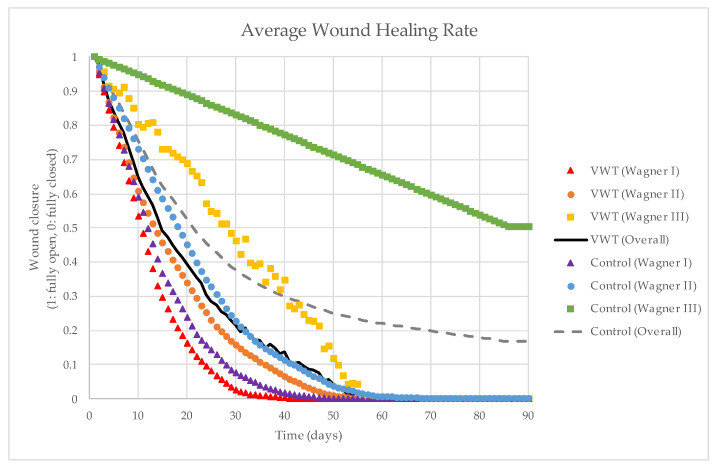
Average wound healing rate for each group and Wagner grade.

**Figure 5:**
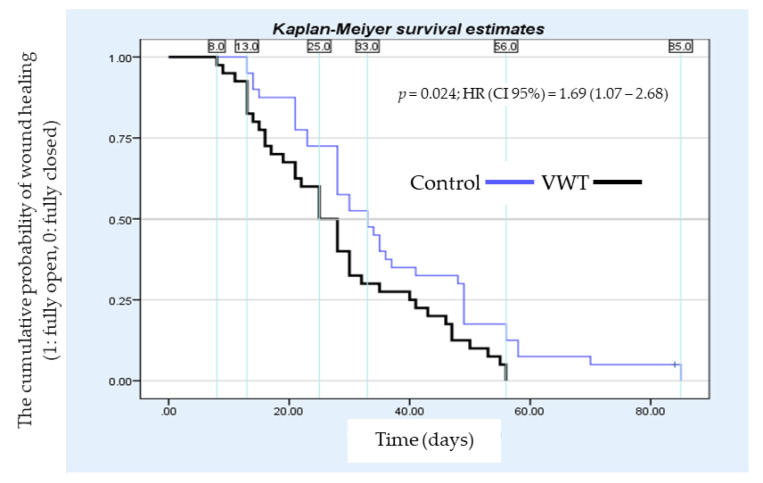
Comparison of the wound healing rate between the intervention and control groups.

In Figure 4, we present the average days needed for the wound to be fully closed for each group and Wagner grade. The average wound closure time for the VWT group was 42, 56, and 58 days, respectively, for Wagner I, II, and III grades of ulcers. On the control group side, the average wound closure time was 49, 70, and 84 days, respectively, for Wagner I, II, and III grades of ulcers. As observed, the wound closure of patients receiving VWT as adjuvant therapy (i.e., intervention group) was consistently faster than the patients in the control group. However, since there were only four patients with Wagner III-grade ulcers in this study, the effect of vibration therapy for Wagner III-grade ulcers needs to be further verified. Nevertheless, overall, we found that the wounds of patients in the intervention group closed faster than the ones in the control group. These differences are clearly presented by the Kaplan–Meier curve in Figure 5.

## 4. Discussion

Diabetic foot problems occur primarily due to underlying neuropathy, peripheral vascular disease, bad glycemic control, or microcirculatory damage. Diabetic foot ulcers are associated with morbidity, extensive care, and healthcare cost. Unfortunately, the precise pathophysiological mechanisms of diabetic foot problems have not yet been elucidated [36]. Considering the previous studies and underlying theories, the most plausible explanation for the accelerated healing rate of diabetic foot ulcers following treatment with VWT is that VWT promotes blood supply and circulation. This result is in accordance with study [26] on rat models with diabetic foot wounds. Study [26] revealed that there is improved circulation in the rat group with vibration intervention. Study [26] also indicated an increased VEGF in the vibration group compared to the control group. As mentioned above, VEGF catalyzes hematopoiesis, bone formation, wound healing, and angiogenesis. Consequently, a faster wound healing rate and better skin microcirculation with upregulation of healing markers were found in the intervention group. Studies using the rat model [26,27,28] conclude that vibration therapy accelerated the wound healing rate of diabetic ulcers. Furthermore, because vibration-induced shear stress causes endothelial cells to release more NO, adjacent vascular smooth muscle cells dilate blood vessels. This vasodilatory effect could improve microcirculation in tissue and promote wound healing [32,43]. A number of studies have found a link between NO productions or NO synthase (NOS) expression and mechanical stress caused by flow stress or exercise [32,34,36,43]. Diabetic patients typically suffer from decreased NO bioavailability, which results in damaged endothelial cells of blood vessels.

A previous study has indicated that the application of 30 Hz vibration therapy for 3 s significantly increased the skin blood flow in the lower extremities [44]. It is known that several factors influence increased blood flow. For instance, extrinsic and intrinsic factors regulate blood flow. Extrinsic factors, including humeral factors and sympathetic nerves originating from external blood vessels, are responsible for controlling the human artery’s blood pressure. On the other side, intrinsic factors act as a regulator for local blood flow to the tissue. Among the intrinsic factors are local metabolism products originating from endothelial cells and vascular smooth muscles.

The previous study shows that muscle exercises can be improved via vibration through local blood flow or stimulation [44]. Therefore, this finding supports the theory that local factors affect peripheral blood flow. Intrinsic factors that stimulate blood vessel vasodilation include NO, prostacyclin (PGI2), and endothelium-derived hyperpolarization factor (EDHF) [45].

Shear stress is among the factors of the mechanisms to release the abovementioned metabolites. Therefore, it is possible that the endothelial cells considered vibration as shear stress and, in turn, increased the production of these factors. Furthermore, studies of shear stress on experimental animals and research at the cellular level indicated that NO plays a vital role in the vasodilation of smooth muscle as a result of shear stress [46].

A decrease in the percentage of NO levels induced additional challenges in the inflammation stage of wound healing, thereby delaying the healing process. Works in the literature have supported the fact that endothelial cells of patients with diabetes are not as responsive as healthy ones toward shear stress. In addition, aging and long-term diabetes will decrease NOS or diminish NO bioavailability [42]. Therefore, it is essential to evaluate whether vibration can enhance NOS and the bioavailability of NO, which, in turn, accelerate the healing of DFU through the increased peripheral blood flow.

The results of this research are supported by the finding of the previous study that at an appropriate magnitude and frequency, vibration is perceived by the human body as mechanical power that stimulates the blood vessels’ endothelial cells to release NO [47]. As mentioned above, NO is a known vasodilator that plays a vital role in improving blood flow and accelerating wound healing. The findings of our study were in line with those of previous studies, which reported that vibration therapy could increase the blood flow of human adults, regardless of their health status (i.e., healthy or suffering from type II diabetes mellitus) [43,44]. The improved blood flow is caused by the increased NOS level triggered by vibration intervention.

In addition, the findings of this study are supported by the previous study [48], which fits into the basic mechanism of blood flow. In the mentioned study, a vibration intervention was applied to mice. The study showed that vibration increased the vasodilation of blood vessels via two mechanisms. First, the synthetic vibration applied to the tissue surface acted as a stress mechanism, resulting in the compression and elongation of endothelial cells. Second, the vibration triggered the stimulating impulse via the polymodalities receptor. This is because polymodality receptors in human subjects are distributed on the skin surface. Hence, *P* substance and calcitonin gene-related peptide were released, causing blood vessel dilatation. Several vibration intensities were applied in our study: 600, 800, and 1000 mVpp, with the vibration frequency maintained at 47 Hz. As soon as 5 and 15 min after the vibration, the blood flow, blood viscosity, and blood vessels were measured. The results indicated an increased blood flow to the skin. The vasodilation of blood vessels became an important basis of wound healing. Nevertheless, we believe that more clinical evidence is required to verify and generalize our findings.

Previous studies have evaluated how effective vibration therapy is capable of facilitating the healing of Stage I pressure ulcers in geriatric patients [32]. The mentioned nonrandom, blinded research was conducted on patients with Stage I pressure ulcers who had been hospitalized for a sufficiently long time. The intervention group retrieved standard treatment for pressure ulcers and complementary vibration therapy, while the control group retrieved only standard treatment for pressure ulcers without vibration therapy.

Our findings are consistent with study [35] and our preliminary work [36], suggesting that optimal vibrations accelerate DFU healing by increasing blood circulation. The results of our study were supported by the findings of the study by Ichioka research, which asserts that vibrations significantly improve skin microcirculation [47]. Secondary outcomes in their clinical trials have shown an increase in muscle blood volume in human subjects. The mechanism of action of NO on wound healing, especially chronic wounds such as diabetic foot ulcers, is caused by the disruption of molecular diffusion of NO to the wound area. This phenomenon is due to the decrease in NO levels in patients with diabetes mellitus caused by interference of the sorbitol pathway.

This research was conducted as a controlled experimental trial, where subjects with diabetic neuropathic foot ulcers were given a combination of standard wound care and therapeutic vibration. One of the goals of this study was to determine changes in the NO levels in patients with diabetic neuropathic foot ulcers who received conventional wound care and therapeutic vibration. Vibration excitation is expected to increase NO production, which helps repair the blood flow to the extremities, thereby accelerating the wound-healing process. According to the findings, it is validated that VWT increased NO levels. On the other hand, patients within the VWT group achieved a faster wound area reduction in a shorter period of time compared to the control group. Based on this fact, and taking into account previous studies [32,33,34,35,36], the change in NO levels after intervention means that the increased levels of NO positively influenced wound healing.

Nevertheless, a further detailed investigation on how VWT increases NO levels and how NO affects the healing rate still needs to be conducted. Although this study was unable to elucidate the underlying mechanism of this phenomenon, we suspect that the vibrations induced shear stress, which in turn activated various pathways that play a role in the improvement of blood flow. Therefore, further research is required to identify possible mechanisms by which vibration influences the healing process of diabetic neuropathy foot ulcers.

In summary, the results of our study suggested that VWT accelerates diabetic neuropathy foot ulcers healing in patients diagnosed with diabetes. This conclusion is derived based on the fact that the patients induced with VWT consistently require a significantly shorter hospitalization time than those who are not. Furthermore, considering that no clinically serious negative effects were found in the patients with VWT intervention, VWT can be regarded as a complementary therapy to the existing ones to accelerate the healing rate of diabetic neuropathy foot ulcers. Furthermore, reducing wound care time and decreasing the healthcare cost will surely help improve the quality of life of patients suffering from diabetic neuropathy foot ulcers. Finally, the consistent findings in this study contribute to the existing literature on vibration therapy’s effect in accelerating wound healing by providing clinical proof and experimental validations.

## 5. Conclusions

In summary, the results of our study suggested that VWT accelerates diabetic neu-ropathy foot ulcers healing in patients diagnosed with diabetes. This conclusion is de-rived based on the fact that the patients induced with VWT consistently require a signifi-cantly shorter hospitalization time than those who are not. Furthermore, considering that no clinically serious negative effects were found in the patients with VWT intervention, VWT can be regarded as a complementary therapy to the existing ones to accelerate the healing rate of diabetic neuropathy foot ulcers. Furthermore, reducing wound care time and decreasing the healthcare cost will surely help improve the quality of life of patients suffering from diabetic neuropathy foot ulcers. Finally, the consistent findings in this study contribute to the existing literature on vibration therapy’s effect in accelerating wound healing by providing clinical proof and experimental validations.

## Study Limitations and Recommendations for Future Works

In this study, the participants were enrolled at different periods of time, making it challenging to blind the group assignment for those assessing the outcomes. While we set the amplitude modulation cycle and the vibrator frequency to 1.78 m/s2 and 47 Hz, respectively, it was difficult to measure the actual intensity and frequency of the vibrator retrieved by each patient in the clinical setting. Further, we did not investigate other combinations of the vibrator’s magnitude and frequency nor the interval and therapy duration variation. Hence, a further study investigating these parameters is needed. The reliability of the healing evaluations was not assessed by a quantitative method prior to the study. In addition, the exact wound area measurement was conducted manually. Although we tried our best to measure the wound area accurately, manual measurement, especially in different periods, is subject to human error. We took into consideration and eliminated other factors that might obscure the outcome, such as additional medical intervention and drug intake (other than the designated ones). The results of this study are in accordance with the underlying theory, as well as previous similar studies on animal and human subjects. However, it is possible that unknown, uncontrollable factors affected the outcome of this study.

We only included patients that met our inclusion criteria. One of the inclusion criteria was neuropathic foot ulcers. Hence, we excluded patients with ischemic foot ulcers. However, it is known that patients with ischemic diabetic foot ulcers have more severe clinical and ulcer appearances as well as poorer outcomes than those with neuropathic diabetic foot ulcers. In addition, ischemic ulcers are the ones that progress toward amputation most of the time [49]. Therefore, in future studies, it is desirable to investigate the effect of vibration therapy on patients with mild-to-medium ischemic ulcers. In this study, we enrolled the participants from two hospitals in one province in Indonesia. Even though this study enrolled only 80 participants, the results are consistent and in line with former studies. However, further study with larger participants from more heterogeneous backgrounds and with a wider yet proportional distribution of Wagner’s classification grades is needed to provide a generalized result. Although we can explain our findings by correlating them with the underlying theories, we did not conduct adequate laboratory analyses to justify our explanation. In the future, it may be possible to investigate the exact mechanism behind the vibration effect on human metabolism and NOS as well as to investigate the effects of NO on wound healing. It is also possible to expand the current study to a broader subject, such as younger patients or patients with arteriosclerosis.

## Figures and Tables

**Table 1:** Characteristics of patients who participated in this study.

Variable	Category	**Control** (*n* = 40)	**Intervention** (*n* = 40)	*p*-Value
Age (years)		56.60 ± 9.03 *	55.20 ± 8.39 *	0.765
Sex	Male Female	13 (32.5%) 27 (67.5%)	16 (40.0%) 24 (60.0%)	0.642
Wound history	No Yes	13 (32.5%) 27 (67.5%)	12 (30.0%) 28 (70.0%)	0.311
Amputation history	No Yes	33 (82.5%) 7 (17.5%)	37 (92.5%) 3 (7.5%)	0.311
Diabetes duration (years)		5.50 (1.00–20.00) ^#^	7.00 (1.00–30.00) ^#^	0.819
Wound duration (weeks)		2.00 (1.00–4.00) ^#^	2.00 (1.00–6.00) ^#^	0.807
BMI (kg/m²)		24.25 ± 3.78 *	24.12 ± 2.83 *	0.630
Wound location	Digit Midfoot Dorsal Midfoot Plantar Others	16 (40%) 7 (17.5%) 14 (35%) 3 (7.5%)	12 (30%) 10 (25%) 13 (32.5%) 5 (12.5%)	0.835
Ulcer’s Wagner Grade	I	17	17	1.000
	II	21	21	
	III	2	2	
Ulcer area (median; CI 95%. mm²)		27.75 (20.02–237.46)	33.99 (20.02–314.39)	0.264
ABI		0.92 (0.81–1083.00)	0.95 (0.75–1076.00)	0.370
Monofilament test	Negative Positive	10 (25.0%) 30 (75.0%)	9 (22.5%) 31 (77.5%)	1.000
HbA1c (%)		9.47 ± 2.58 *	9.12 ± 2.16 *	0.538
Albumin		3.38 ± 0.64 *	3.46 ± 0.66 *	0.722
NO level		1.12 (0.19–4.84) ^#^	0.92 (0.03–2.97) ^#^	0.502

Data are the val. ^(#)^ Median, Cox regression. Values are either *n* or median ± confidence intervals (*); (min–max) indicates the minimum and the maximum observed values.

**Table 2:** Diabetic wound healing rate.

Group	n	n **Healed**	Time Coeff.	Median (Days) (CI 95%)	Incident Rate (Healing)	*p*-Value	HR	HR 95% CI Min—Max	
VWT	40	40	1.144	25 (20.3–29.7)	0.035	0.024	1.69	1.07	2.68
Control	40	39	1.471	33 (25.6–40.4)	0.027				

**Table 3:** Effect size of diabetic wound healing rate.

Group	n	Median (Days) Min–Max		Effect Size *r*	Cohen’s *d*	Glass’s Δ	Hedges’ *g*
VWT	40	20.3	29.7	0.810	2.764	2.311	2.772
Control	40	25.6	40.4				

**Table 4:** Wound closure area between groups.

Mann–Whitney Test	n	Median	Min–Max	*p*-Value
Relative wound area reduction (%/day)				
Control	40	3.03	(−0.01)–7.69	0.032
VWT	40	3.79	1.79–12.50	
Delta wound area reduction (mm/day)				
Control	40	1.01	(−0.01)–5.56	0.021
VWT			0.45–18.55	

**Table 5:** Differences in NO levels between and within groups, before and after the interv.ntion.

Group	Week 0	Week 12	*p*-Value	Delta
Control	1.12 (0.19–4.84)	0.86 (0.36–2.60)	0.065	((−4.16)–2.60)
VWT	0.92 (0.03–2.97)	2.91 (0.96–6.60)	<0.001	1.39 (0.10–6.60)
*p*-Value	0.502	< **0.001**		< **0.001**

## Data Availability

Data are available from the corresponding author upon reasonable request.
